# Changes in the brain structural connectome after a prospective randomized clinical trial of lithium and quetiapine treatment in youth with bipolar disorder

**DOI:** 10.1038/s41386-021-00989-5

**Published:** 2021-03-22

**Authors:** Du Lei, Wenbin Li, Maxwell J. Tallman, L. Rodrigo Patino, Robert K. McNamara, Jeffrey R. Strawn, Christina C. Klein, Fabiano G. Nery, David E. Fleck, Kun Qin, Yuan Ai, Jing Yang, Wenjing Zhang, Su Lui, Qiyong Gong, Caleb M. Adler, John A. Sweeney, Melissa P. DelBello

**Affiliations:** 1 Department of Psychiatry and Behavioral Neuroscience, University of Cincinnati College of Medicine, Cincinnati, OH USA; 2 Huaxi MR Research Center (HMRRC), Department of Radiology, The Center for Medical Imaging, West China Hospital of Sichuan University, Chengdu, P. R. China

**Keywords:** Bipolar disorder, Outcomes research

## Abstract

The goals of the current study were to determine whether topological organization of brain structural networks is altered in youth with bipolar disorder, whether such alterations predict treatment outcomes, and whether they are normalized by treatment. Youth with bipolar disorder were randomized to double-blind treatment with quetiapine or lithium and assessed weekly. High-resolution MRI images were collected from children and adolescents with bipolar disorder who were experiencing a mixed or manic episode (*n* = 100) and healthy youth (*n* = 63). Brain networks were constructed based on the similarity of morphological features across regions and analyzed using graph theory approaches. We tested for pretreatment anatomical differences between bipolar and healthy youth and for changes in neuroanatomic network metrics following treatment in the youth with bipolar disorder. Youth with bipolar disorder showed significantly increased clustering coefficient (*C*_*p*_) (*p* = 0.009) and characteristic path length (*L*_*p*_) (*p* = 0.04) at baseline, and altered nodal centralities in insula, inferior frontal gyrus, and supplementary motor area. *C*_*p*_, *L*_*p*_, and nodal centrality of the insula exhibited normalization in patients following treatment. Changes in these neuroanatomic parameters were correlated with improvement in manic symptoms but did not differ between the two drug therapies. Baseline structural network matrices significantly differentiated medication responders and non-responders with 80% accuracy. These findings demonstrate that both global and nodal structural network features are altered in early course bipolar disorder, and that pretreatment alterations in neuroanatomic features predicted treatment outcome and were reduced by treatment. Similar connectome normalization with lithium and quetiapine suggests that the connectome changes are a downstream effect of both therapies that is related to their clinical efficacy.

## Introduction

Bipolar disorder is a common debilitating illness that often has an onset during adolescence [1–3]. Studying adolescents with bipolar disorder provides a window of opportunity for clarifying illness neurobiology close to the time of onset, and for identifying biological alterations that predict and track clinical change after treatment in individuals with limited prior medication exposure.

Functional and structural imaging with MRI has been useful not only for understanding illness biology, but for evaluating medication effects and predicting treatment response [4, 5]. While anatomy has generally been considered to be relatively static in adult life, recent evidence indicates that both behavioral training and acute treatment with psychotropic medications measurably alter gray matter structures. This includes studies of antipsychotics and lithium in patients with schizophrenia and bipolar disorder [6–8]. Recently, seed-based functional connectivity has been employed to predict treatment response in patients with bipolar disorder treated with mood stabilizers [9]. Moreover, evidence suggests that the pathophysiology of bipolar disorder involves network dysconnectivity [10–12], so that examining the brain connectome may be a promising strategy for advancing illness understanding and biomarker development.

Graph-based analysis provides a powerful tool for characterizing topological properties of brain networks (i.e., the connectome) [13, 14] and their alteration in psychiatric disorders [15, 16]. In this approach, the brain is modeled as a network composed of a number of nodes and edges connecting the nodes. Nodes represent individual cortical and subcortical regions, and the edges reflect their connectivity, which is needed for the transfer of information among these regions that serves as the basis for higher brain function. Based on graph theoretical analysis, the integrity of the structural connectivity of the brain can be represented by a series of network measures that quantify the segregation and integration features of network topology. Recently, high-resolution structural MRI has been increasingly used to delineate whole-brain connectivity patterns by evaluating patterns of interregional gray matter (GM) volume measurements [17–20]. Such measures can help to understand how alterations in network anatomy shape functional and behavioral characteristics, such as those associated with bipolar disorder [21, 22].

In addition to studying illness mechanisms, this approach can also be used to identify biomarkers that predict treatment response and to determine how widely used therapeutics impact the brain connectome. Lithium is one of the oldest and most effective treatments for mania [23, 24]. Quetiapine is also efficacious for manic episodes, and in some manic adolescents may be a more effective therapy than widely used mood stabilizers [25, 26]. Although investigators have proposed models for understanding the therapeutic effects of lithium in bipolar disorder [27–33], there are few studies of the in vivo effects of lithium or quetiapine on markers of gray matter network properties in manic patients.

We conducted a randomized placebo-controlled clinical trial in youth with bipolar disorder and the graph-based approach was used to characterize gray matter connectome metrics. Our aims were to conduct a case-control comparison to evaluate structural connectome alterations in bipolar youth, to determine the effects of lithium and quetiapine on GM networks in a randomized clinical trial, and to determine if baseline metrics could predict treatment response. First, since previous fMRI studies have found the network abnormalities in the salience network (SN) in the medication-naïve and medication-free BD patients [34], we hypothesized that youth with bipolar disorder also would display pretreatment GM brain network abnormalities at both global and regional level compared with healthy comparison youths especially in the SN. Second, since previous structural MRI study didn’t find significant differences of longitudinal treatment effects between lithium and quetiapine group in terms of gray matter volume [35], we do not hypothesize lithium and quetiapine would have a different treatment impact on the GM network proprieties, though of course we did explore that possibility. Given that both lithium and quetiapine effects on brain anatomy and function are reported in previous studies [8, 36, 37], we hypothesized that six weeks of lithium or quetiapine treatment would elicit changes in the global topological metrics as well as regional nodal centralities of key brain region of SN network (including insula and anterior cingulate cortex), and that these changes would be related to reductions in mania symptom severity. Third, we predicted that GM network matrices prior to treatment would predict mania symptom reduction after treatment.

## Materials and methods

### Participants

The University of Cincinnati and the Cincinnati Children’s Hospital Medical Center Institutional Review Boards approved this study. All study participants and their legal guardians provided written informed assent and consent, respectively. A total of 100 youth with bipolar I disorder (*DSM-IV-TR* criteria), and 63 healthy comparison subjects were included. Participants were recruited from the Cincinnati Children’s Hospital Medical Center, the University of Cincinnati Medical Center, and the local community. A diagnosis of bipolar I disorder was confirmed using the Washington University in St. Louis Kiddie Schedule of Affective Disorders and Schizophrenia (WASH-U-KSADS) [38] administered by raters with demonstrated inter-rater diagnostic reliability (*kappa* > 0.9). Psychiatric symptoms were rated using the Young Mania Rating Scale (YMRS) [39], Children’s Depression Rating Scale-Revised (CDRS-R) [40], and the Clinical Global Impressions-Severity Scale (CGI-S) [41] (*ICC* > 0.7). Parental socioeconomic status (SES) was evaluated using the Hollingshead–Redlich scale [42].

Participants were included who met the following criteria: (1) within the age range of 10–17 years and 11 months old, (2) experiencing a manic or mixed episode, (3) having a baseline YMRS score ≥ 20, (4) within 2 years from onset of bipolar disorder, (5) having no prior psychiatric hospitalizations for mania, and (6) having no history of treatment with therapeutic doses of antipsychotics or mood stabilizers for > 3 months, and no psychotropic medication during the week (72 h for psychostimulants) prior to the baseline scans. Patients could have prior attention deficit and hyperactivity disorder treatment and up to 3 months of prior antidepressant treatment, since excluding these patients would significantly limit the generalizability of study findings. Demographically matched healthy youths were recruited from the communities in which the bipolar participants resided, and were screened to establish the lifetime absence of mood or psychotic disorder among their first- or second-degree relatives using the Family Interview for Genetics Studies (FIGS). All participants were Tanner stage III–V [43] in order to exclude prepubertal subjects and minimize the effects of brain changes associated with pubertal onset [44].

Participants were excluded if having: (1) a contraindication to MRI scanning (e.g., braces or claustrophobia); (2) an IQ < 70, as determined by the Wechsler Abbreviated Scale of Intelligence [45]; (3) a positive pregnancy test; (4) a history of a major systemic or neurological illness, or an episode of loss of consciousness > 10 min; (5) any lifetime *DSM-IV-TR* substance use disorder (nicotine dependence was permitted); or (6) a lifetime *DSM-IV-TR* diagnosis of a pervasive developmental disorder or post-traumatic stress disorder (PTSD). Participants were enrolled in the study by a research coordinator (CCK) if they passed screening of these inclusion/exclusion criteria.

### Treatment procedures

Following a clinical evaluation and MRI scanning, patients were randomized, by an investigational pharmacist, to double-blind treatment with quetiapine or lithium, and evaluated weekly for 6 weeks. The randomization schedule was stratified by presence/absence of attention deficit and hyperactivity disorder, presence/absence of psychosis, and the mood state (mixed vs. manic episode). Treatment response was defined as a ≥ 50% reduction in YMRS scores from baseline [9, 46]. Study coordinators, psychiatrists, and participants were blinded to the treatment group, except for an un-blinded psychiatrist monitoring dosage of medications, as described in the treatment procedures section presented in Supplemental Information. No other concomitant psychotropic medications were permitted during the course of the study (including antidepressant and ADHD medications).

### Data acquisition

MRI scans were performed using a 4-T Varian Unity INOVA scanner with a 12-channel head coil at baseline (prior to treatment) and again at weeks 1 and 6. Following a three-plane gradient echo scan for alignment and localization, a shim procedure was performed to generate a homogeneous magnetic field. High-resolution T1-weighted three-dimensional images were acquired with a Modified Driven Equilibrium Fourier Transform (MDEFT) protocol, optimized for the 4T Varian scanner (T_au_ (magnetization preparation time) = 1.1 s, TR = 13 ms, TE = 5.3 ms, field of view = 192 × 256 × 256 mm, matrix = 192 × 256 × 256, flip angle = 20 degrees, slice thickness = 1 mm. T1-weighted images were inspected by two experienced neuroradiologists who made decisions about excessive motion artifact for scan inclusion. No observable scanning artifacts or gross brain abnormalities were identified in any participant included in analyses.

### MRI data preprocessing

Structural images were processed using Statistical Parametric Mapping software (SPM12; http://www.fil.ion.ucl.ac.uk/spm). In brief, individual structural images were first segmented into GM and white matter (WM) using the unified segmentation model [47]. The resulting GM maps were then normalized to the Montreal Neurological Institute (MNI) space using a high-dimensional “Diffeomorphic Anatomical Registration Through Exponentiated Lie Algebra (DARTEL)” approach and subjected to nonlinear modulation to compensate for spatial normalization effects. Finally, the GM data were re-sampled to 1.5 mm^3^ voxels and spatially smoothed (Gaussian kernel with a full width at half maximum of 6 mm).

### Construction of GM structural networks

In a network analysis, nodes are the most basic units, and the network is constituted by the nodes and relationships between them. The relationships between nodes are called edges in network analysis. In this study, we used automated anatomical labeling (AAL) template that divides the cerebral cortex and subcortical structures into 90 independent anatomical regions [48]. A well-established method which called Kullback–Leibler divergence-based similarity (KLS) was applied to characterize the connections or edges between anatomic regions [49, 50]. In short, the higher similarity of gray matter density distribution between two anatomical regions, the higher KLS scores between them and then stronger connections and shorter edges between them. The range of KLS is from 0 to 1, with 1 representing an identical density distribution for two regions. We calculated the KLS values between all possible pairs of 90 brain regions and thus a 90 × 90 similarity matrix for each subject was generated. In this 90 × 90 network matrix, each row and column represent a brain region and each element represents the similarity of morphological distributions between a pair of brain regions.

### Network properties

The GRETNA toolbox (http://www.nitrc.org/projects/gretna/) was used to calculate network properties of brain networks. We applied a wide range of sparsity (*S*) thresholds to all correlation matrices. The value of *S* was chosen to ensure that thresholded networks were estimable for the small-worldness scalar and the small-world index (σ) was larger than 1.0 [51]. The range of our *S* thresholds was set to 0.10 < *S* < 0.34 with an interval of 0.01. For each network metric, the area under the curve (AUC) was calculated, which provides a summarized scalar for the topological characterization of brain networks independent of a single threshold selection. The AUC metric has proven to be sensitive for the detection of topological alterations of brain networks [52, 53].

Both global and nodal network properties were calculated for brain networks at each sparsity threshold. Global metrics, including small-world [51] and network efficiency parameters [54], were examined. The small-world parameters included clustering coefficient (*C*_*p*_), characteristic path length (*L*_*p*_), normalized clustering coefficient (γ), normalized characteristic path length (λ), and small worldness (σ). Network efficiency parameters included local efficiency (*E*_loc_) and global efficiency (*E*_glob_). Metrics pertaining to individual nodes, including nodal degree, nodal efficiency, and betweenness centrality, were also examined. The brief definition and description of the topological measures used in the current study and more discussion of feature interpretation are available can be found in supplementary materials.

### Statistical analysis

To identify significant between-group differences in network properties, nonparametric permutation tests [53] were performed on the AUC of each network metric. To test null hypotheses, we randomly reallocated all values for each network metric into two groups and recomputed the mean differences between them. This randomization procedure was repeated 10,000 times, and the 95^th^ percentile points of each distribution were used as the critical values for a two-tailed test of the null hypothesis with a type I error of 0.05.

Regression analyses were conducted in *R* software (version 3.5.3). Group-by-time interaction effects were examined for all global and nodal metrics that differed between patients with bipolar disorder and healthy controls at baseline, using mixed effects models including data from all three time points. We also investigated relations between changes in network measurements from baseline to endpoint with changes in clinical symptom scores (i.e., YMRS). Age and sex were treated as covariates in these models. In order to assess medication-specific differences between quetiapine and lithium on topological alterations, treatment-by-time interactions were also examined using mixed effects models. All global and nodal measurements that differed between treatment groups at baseline were examined across all three time points.

Finally, as an additional strategy for examining whether baseline structural connectome measures predict treatment response after 6 weeks of treatment, we applied support vector machine (SVM) to the baseline GM morphological matrices (90 × 90 Pearson correlation matrix) to classify responders vs. non-responders (The prediction is considered for completers only). Statistical significance was estimated using the permutation method (1,000 permutations). To obtain a reliable estimate of the performance of the models, we used a 10-fold stratified cross-validation scheme. This model was described in detail in Supplemental Materials.

## Results

### Demographic and clinical characteristics

There were no significant statistical differences in age [mean (standard deviation): 14.6 (2.0) vs. 14.9 (1.9), *p* = 0.29] or sex [Sex Male (%): 39% vs. 43%, *p* = 0.62] between the patient and healthy comparisons groups nor between treatment groups [age: 15.2 (2.0) vs. 14.3 (1.5), *p* = 0.05; sex: 36% vs. 40%, *p* = 0.65]. There were no significant differences in clinical measures between the lithium and quetiapine treatment groups at baseline. In both treatment groups, YMRS and CDRS-R scores decreased over time from baseline to week 6 (*p* < 0.001) (Supplemental Information, Table S1). The distribution of YMRS at baseline and changes are provided in the supplemental information (Fig. S1).

### Alterations of brain network properties at baseline

**Fig. 1:**
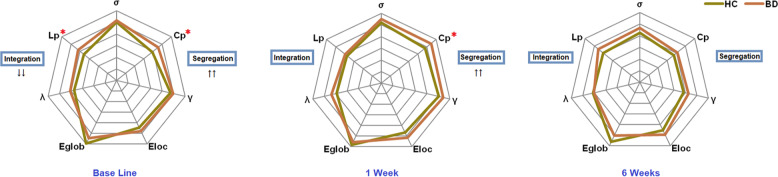
Graphs show differences in global topological properties between patients with bipolar disorder and health controls at different time points. The clustering coefficient (*C*_*p*_) (*p* = 0.009) and characteristic path length (*L*_*p*_) (*p* = 0.04) were significantly different between the two groups at the baseline. Increased *C*_*p*_ was still significantly different (*p* = 0.045) after 1 week’s treatment. An asterisk designates network metrics with a significant difference (*p* < 0.05).

For global measurements, six out of all seven global topological metrics (except λ) showed at least fair (fair, good, and excellent) intraclass correlation coefficients (*ICC* > 0.4); for nodal measurements, 82.6% centralities showed above fair *ICC* in the HC group based on Cicchetti’s guidelines [55] (Table S2). In the defined threshold range, both the patient and healthy comparison groups showed small-world topology in brain structural connectomes at baseline but patients had increased *C*_*p*_ (*p* = 0.009) and *L*_*p*_ (*p* = 0.04) (Fig. 1).

**Fig. 2:**
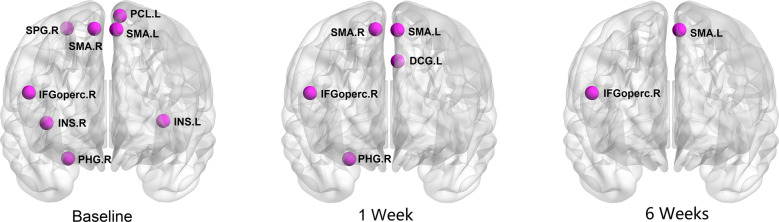
Graphs show the regions with significantly altered nodal centralities of the brain structural connectome in patients with bipolar disorder when compared with health controls at different time points. The nodes were mapped onto the cortical surfaces by using the BrainNet Viewer package (http://www.nitrc.org/projects/bnv). DCG median cingulate and paracingulate gyri; IFGoperc inferior frontal gyrus, opercular part; INS insula; PCL paracentral lobule; area; PHG parahippocampal gyrus; SMA supplementary motor area; SPG superior parietal gyrus. R right hemisphere; L left hemisphere.

Brain regions exhibiting significant between-group differences in at least two out of three nodal metrics of topological centrality were identified. Such differences were seen in multiple areas of heteromodal cortex including right inferior frontal gyrus, bilateral supplementary motor area, bilateral insula, right parahippocampal gyrus, right superior parietal gyrus and left paracentral lobule (Fig. 2 and Table 1). At baseline, there were no significant differences in any network parameters between the two treatment groups.

**Table 1:** Regions showing changed nodal centralities in the bipolar disorder group compared with health control group across time points.

		*P* values	
Brain regions	Nodal degree	Nodal efficiency	Nodal betweenness
Baseline			
Right inferior frontal gyrus (opercular part)	**0.010**	0.151	**0.008**
Left supplementary motor area	**0.006**	**0.004**	0.341
Right supplementary motor area	**0.046**	**0.044**	0.580
Left insula	**0.016**	**0.017**	0.631
Right insula	**0.019**	**0.045**	0.585
Right parahippocampal gyrus	**0.024**	0.051	**0.033**
Right superior parietal gyrus	0.117	**0.039**	**0.048**
Left paracentral lobule	**0.019**	**0.007**	**0.017**
1 Week			
Right inferior frontal gyrus (opercular part)	**0.014**	0.138	**0.020**
Left supplementary motor area	**0.001**	**0.001**	0.457
Right supplementary motor area	**0.019**	**0.019**	0.354
Left median cingulate and paracingulate gyri	**0.025**	**0.048**	0.175
Right parahippocampal gyrus	**0.031**	**0.041**	0.134
6 Weeks			
Right inferior frontal gyrus (opercular part)	**0.044**	0.215	**0.028**
Left supplementary motor area	**0.023**	**0.015**	0.966

Regions were considered abnormal in the bipolar disorder group if they exhibited significant between-group differences (*P* < 0.05, uncorrected) in at least two of the three nodal centralities (shown in bold font). All the brain regions are from automated anatomical labeling (AAL).

### Changes in topological alterations after medication treatment

The nodal degree and nodal efficiency of the left parahippocampal gyrus showed a significant treatment-by-time interaction (*F* = 3.94, *p* = 0.048 and *F* = 3.92, *p* = 0.049) and nodal betweenness of the left precentral gyrus showed a significant treatment-by-responder interaction (*F* = 4.54, *p* = 0.03), suggesting differences in treatment effects between the two treatment groups. However, these results were not statistically significant after FDR correction for multiple comparisons. Therefore, in further analyses of changes after treatment and their correlations, the two treatment groups were combined.

Following treatment initiation, patients still showed increased *C*_*p*_ (*p* = 0.045) at week 1 relative to controls, but there were no statistically significant differences in global brain network properties between patient and control groups at week 6 (Fig. 1). For the significant nodal network alterations observed at week 1, bipolar patients exhibited fewer differences when compared to healthy comparison subjects than at baseline, but alterations were still seen in right inferior frontal gyrus, bilateral supplementary motor area, left median cingulate and paracingulate gyri, and right parahippocampal gyrus. At week 6, only the right inferior frontal gyrus and left supplementary motor area showed nodal alterations when compared to healthy controls (Fig. 2 and Table 1).

**Fig. 3:**
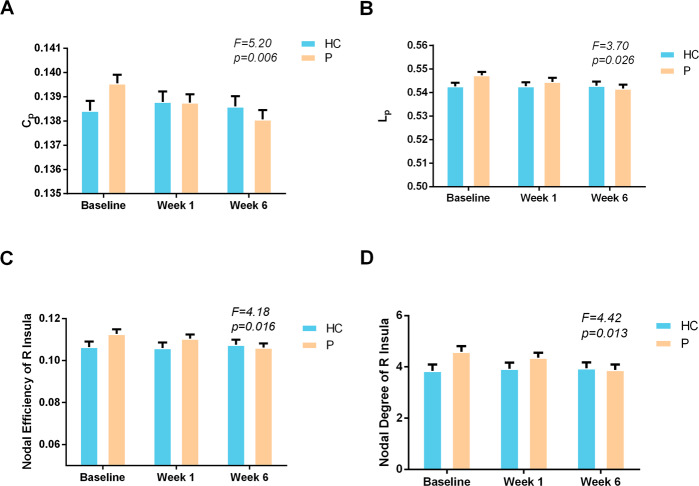
Network parameters that changed over the course of treatment in bipolar patients. Significant group-by-time effects were observed in both global level in C_*p*_ and L_*p*_ (**A** and **B**) and regional level in nodal efficiency and degree of right insula (**C** and **D**). *F* values of each significant time by group interaction are provided.

*C*_*p*_ (*F* = 5.2, *p* = 0.006), *L*_*p*_ (*F* = 3.7, *p* = 0.026) and nodal efficiency (*F* = 4.18, *p* = 0.016) and nodal degree (*F* = 4.42, *p* = 0.013) of the right insula showed significant group-by-time interaction effects (Fig. 3). For all these measures, the patients showed normalization in parameter estimates while controls remained unchanged.

### Correlation of network alterations with clinical symptom severity

**Fig. 4:**
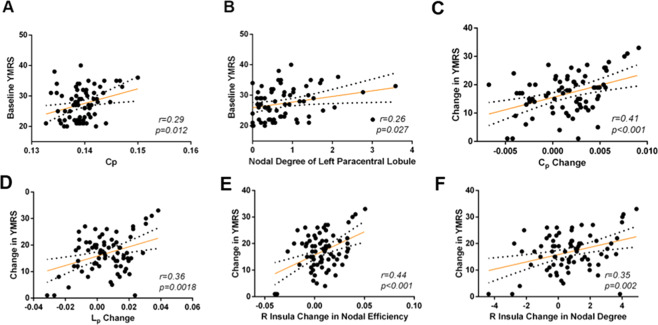
Graphs show correlations of change in network metrics with change in clinical symptom severity. **A**, **B** show relations of network metrics to symptom severity at baseline. **C**–**F** show relations between change of clinical symptom severity and normalization of altered network metrics at week 6. Abbreviations: SMA = supplementary motor area.

At baseline, we did not detect significant correlations between altered network properties and age, IQ or SES. In patients, *C*_*p*_ (*r* = 0.29, *p* = 0.012, Fig. 4A) and nodal degree of the left paracentral lobule (*r* = 0.26, *p* = 0.027, Fig. 4B) were positively correlated with YMRS scores. At week 1, changes in network metrics were not significantly correlated to changes in YMRS ratings.

Reductions in C_p_ (baseline to week 6) were correlated with YMRS reductions from baseline to week 6 (*r* = 0.41, *p* < 0.001, Fig. 4C), as were changes in L_p_ (*r* = 0.36, *p* = 0.0018, Fig. 4D). Reductions in nodal efficiency and nodal degree of the right insula were significantly correlated with YMRS scores changes (*r* = 0.44, *p* < 0.001, Fig. 4E and *r* = 0.35, *p* = 0.002; Fig. 4F, respectively). These results were statistically significant even after FDR correction for multiple comparisons. We did not detect significant correlations between altered network properties and CDRS-R score changes.

### Machine learning analysis applying baseline GM morphological network matrices to predict treatment response

Using GM morphological matrices, the mean balanced accuracy of classification for predicting treatment response was 75% for the lithium group, 85% for the quetiapine group and 82% for all bipolar patients combined (*p* < 0.001), which are well above chance expectation. Following up on this finding, we identified the regions contributing to successful classification performance. We computed the mean absolute value of the weights of the model across the different folds of the cross-validation. For each nodal region, the mean value of the weights for its structural connectivity with the remaining 89 regions was calculated. The top 10 brain region measurements at baseline with the highest mean values in relation to clinical outcome were extracted. We found that insula was among the top brain region contributing to treatment outcome prediction for both treatment groups, consistent with our study hypothesis. Full information of brain regions contributing to the SVM classification is reported in Table S3 of supplemental material.

## Discussion

To our knowledge, this is the first study to apply graph theory-based analytic methods to structural MRI images in an effort to identify abnormalities in topological properties of GM networks that predict and track with treatment outcomes in youth with bipolar disorder. This was achieved using recently developed methods for describing patterns of intracortical similarities of nodes in the brain network. We found that youth with bipolar disorder showed altered structural connectome metrics at baseline, and these alternations exhibited normalization in patients after treatment. We demonstrated that pretreatment GM morphological matrices differentiated treatment responders and non-responders with 80% accuracy. This pattern of results indicates a utility for neuroanatomic connectome measures for predicting clinical outcome and detecting drug treatment related effects on brain anatomy in a pediatric bipolar sample.

According to Watts et al [51], networks can be divided into three types. Considering the total number of connections as a constant: (1) If all nodes are only connected to their nearby nodes, and there is no long-range connections, this kind of network is called a regular network (2) If the probabilities of having the short- and long-range connections are the same, this kind of network is called a random network (3) If there are a large number of short-range connections, but also a small number of long-range connections, it is considered as a small-world network. Compared with regular and random networks, the small-world networks guarantee the efficiency of information transfer between local networks while ensuring the integrity within local networks which themselves may hold specific functions. A regular network is characterized by a higher *C*_*p*_ (the probability that neighboring nodes are also interconnected with other neighboring nodes) and a longer *L*_*p*_ (the average distance from one node to any other node in the network, expressed as the number of links that must be traveled) [51]. Youth who were experiencing a mixed or manic episode exhibited a more regularized brain network at baseline, reflected in increased *C*_*p*_ and increased *L*_*p*_, in which the network transforms from a small-world network to a relatively regular network. This pattern of results is consistent with our first hypothesis, and demonstrate alterations in structural brain connectomes in patients with pediatric bipolar disorder. Previous studies applying connectome-based technologies on task-based fMRI [56], resting-state fMRI [57], and structural images [10, 20], found that adult patients with bipolar disorder had decreased integration (increased *L*_*p*_ or decreased *E*_glob_), indicating a shift toward regularization of the network. This form of global network disruption has been previously reported in other neuropsychiatric disorders including in adults with PTSD [58], schizophrenia [59], and major depressive disorder [60]. The increased segregation of brain networks reflected by higher *C*_*p*_ might underlie impaired cognitive and emotional processing in youth with bipolar disorder, as suggested by correlations between *C*_*p*_ and psychiatric symptom ratings (YMRS) in the present study.

At the regional level, analyses of nodal alterations using nominal significance thresholds revealed altered nodal centralities in multiple brain regions at baseline, including the right inferior frontal gyrus, bilateral insula, and bilateral supplementary motor area (SMA). The insula is a major hub [61] within the salience network (SN) [62] and a key structure for integrating cognitive, behavioral, and affective functional processes [63]. The SMA is considered to provide an interface between the emotional system and behavioral planning for the motor system [64, 65]. These abnormal nodal centralities suggest reduced integration of key regions in neocortical anatomic networks relevant to emotion processing.

Because there were no significant differences after FDR correction in any network parameters between the two treatment groups at baseline or in their change over time, we combined the two treatment groups in our primary analyses testing for treatment-related effects. Consistent with our second hypothesis, significant group-by-time interaction effects were found in *C*_*p*_ and *L*_*p*_ after medication treatment, showing similar reductions of abnormalities in both treatment groups relative to retested healthy controls. These findings indicate that following treatment, brain anatomic networks of the bipolar patients reverted from regularization toward a more typical small-world pattern.

Analyses revealed that nodal centrality of the right insula normalized after treatment, and that these changes were significantly associated with changes in mania symptom ratings. Notably, connectome integration abnormalities in this region were reduced by pharmacological treatment within six weeks. Additionally, among the nominally altered nodal centralities at baseline, only the right inferior frontal gyrus and left SMA were still abnormal at endpoint. This finding is important not only for the importance of localized findings but in showing that clinically meaningful changes in brain anatomy can be detected even after acute pharmacotherapy. In the two regions with persistent deficits, nominally abnormal nodal centralities that persisted after treatment indicate a persistence of alterations in these regions which may underlie trait-related alterations that may contribute to ongoing disturbances in emotional and cognitive functions [66].

In the present study, consistent with our third hypothesis, we achieved a significant accuracy in predicting clinical outcome following treatment (82%) using all patients’ GM connectome data. This provides support for the emerging view that network biomarkers have the potential to improve classification and treatment prediction of neuropsychiatric disorders [67, 68], in this case for youth with bipolar disorder. Consistent with our success using network metrics, a recent study suggested that connectome-wide metrics can have greater diagnostic value than whole-brain image data [69]. Our results are consistent with this, suggesting that GM morphological matrices might have high predictive value for medication response.

Neuroimaging is still far from becoming a tool used in the day-to-day clinical practice of psychiatry, though findings from the present study represent a step forward in laying a foundation for such developments. This is because at present there is still insufficient evidence that imaging features can reliably support diagnostic evaluations or treatment planning for psychiatric patients (especially when it comes to differential diagnosis). In this context, our findings not only provide novel insights into the understanding of brain structural network alterations in early bipolar disorder, and the level of utility of structural network alterations for diagnosis, but that these alterations can predict the treatment response. Because such prediction has been challenging using information from clinical psychiatric evaluations, this area may represent one where MRI scans might be particularly useful for psychiatric practice.

In this study, we found that the quetiapine group data allowed a higher accuracy of prediction than in the lithium group (mean 85% vs. 75%). This might be due to the quetiapine group having a bigger sample size than the lithium group. An alternative explanation is that these two medications have different drug mechanisms, MRI data is more useful for outcome prediction in the quetiapine group. Another important observation was that the insula was among the top brain regions contributing predicting treatment outcome for both treatment groups. This is noteworthy because this region has altered nodal properties in patients relative to healthy controls at baseline and is well known to be an important brain region for emotion processing. In contrast, the connectivity of the inferior frontal gyrus was only among the top brain regions contributing to treatment outcome in the quetiapine-treated group. Previous studies have suggested that this region is a key cortical hub in the circuits of emotion and cognitive control, and its alteration in bipolar disorder has been reported [70]. This pattern of results suggests that overlapping but distinct structural connectome alterations may predict lithium and quetiapine treatment response, a possibility that requires further study.

In the present study, exploratory treatment-by-time analyses revealed nominally significant differences in nodal degree and nodal efficiency of the left parahippocampal gyrus between the treatment groups. A previous study reported that lithium treatment in bipolar disorder has a significant effect on brain structure, particularly in limbic/paralimbic regions associated with emotional processing [71]. Little is known about the differential acute effects of quetiapine vs. lithium on the brain structural connectome. Our results failed to identify a statistically difference between these two medications in their overall ability to induce structural changes in youth with bipolar disorder, but this might be explained by the relatively small sample size in each treatment group. As the direct drug mechanisms of lithium and quetiapine are different [72], similarity at the level of global network parameters suggest that connectome changes may be downstream of direct pharmacological drug effects, with both drugs having similar effects on illness-related connectome alterations that were related to reductions in manic symptoms and behavior.

The present study has several limitations. First, the brain parcellation template we selected for constructing brain networks may affect the network analysis results. As different templates may lead to different estimates of graph theory parameters, this factor needs to be considered [73]. Second, the patients in the lithium group (37%) had a higher drop-out rate than in the quetiapine group (16%) at week 6, which may affect our conclusions. Third, additional psychological evaluations are needed in future work to precisely define the aspects of emotional and behavioral features that are related to the observed connectome alterations and their change with treatment. Finally, while the construction of individual structural networks based on the similarity of morphological distributions can be informative about neuroanatomic architectures, the functional implications of these anatomic network alterations need to be more fully understood.

In conclusion, despite the above limitations, the present study demonstrates both global and nodal network alterations in manic youth with bipolar disorder compared to healthy youth. Following treatment, several baseline alterations in the anatomic connectome were significantly reduced. Further, baseline structural connectivity data successfully predicted treatment response after 6 weeks of quetiapine or lithium treatment. These results suggest that measures of the structural connectome may contribute to the prediction of medication treatment outcome in youth with bipolar disorder.

## Funding and disclosures

This study was supported by the National Institute of Mental Health (NIMH) Grant (Grant No. 5R01MH080973 (DelBello)) and the National Natural Science Foundation of China (Grant No. 81621003).

Dr. McNamara has received investigator-initiated research grant support from Martek Biosciences Inc, Royal DSM Nutritional Products, LLC, and Inflammation Research Foundation, and investigator-initiated research support from Ortho-McNeil Janssen, AstraZeneca, Eli Lilly, NARSAD, and national institutes of health (NIH), and previously served on the scientific advisory board of the Inflammation Research Foundation.

Dr. Nery himself reported no conflicts of interests. Nery’s spouse is an employee at Eli Lilly and Co.

Dr. Strawn has received research support from the National Institutes of Health (NIMH/NIEHS/NICHD) as well as Allergan, Neuronetics, and Otsuka. He has received material support from and provided consultation to Myriad Genetics and receives royalties from the publication of two texts (Springer) and serves as an author for UpToDate and an Associate Editor for Current Psychiatry. He has spoken in CME presentations for Neuroscience Education Institute and CMEology. Finally, Dr. Strawn also has provided consultation to the FDA and Intracellular Therapeutics.

Dr. Sweeney consults to VeriSci.

Drs. DelBello and Adler are on the lecture bureau for Otsuka, and Dr. Adler is on the lecture bureau for Janssen. Dr. Patino and Dr. DelBello have received research support from Acadia, Allergan, Janssen, Johnson and Johnson, Lundbeck, Otsuka, Pfizer, Sunovion and Supernus and Dr. DelBello has provided consultation or advisory board services for Alkermes, Allergan, Assurex, CMEology, Janssen, Johnson and Johnson, Lundbeck, Neuronetics, Otsuka, Pfizer, Sunovion and Supernus. Dr. Adler has received research support from Merck, Forest, and Alkermes, and provided consultation for Janssen. All other authors declare that they have no competing interests.

## Supplementary information

Supplemental Information

CONSORT 2010 Flow Diagram
