# Supplementary material for: Changes in the brain structural connectome after a prospective randomized clinical trial of lithium and quetiapine treatment in youth with bipolar disorder
Source: Neuropsychopharmacology. 2021 Mar 22;46(7):1315–23. doi: 10.1038/s41386-021-00989-5 (PMC8134458; doi:10.1038/s41386-021-00989-5)
Supplement: Supplementary file 1 — Supplemental Information [file 41386_2021_989_MOESM1_ESM.docx]

**Supplemental Information**

**Sample size estimation**

We planned to recruit 120 (approximately 2 subjects/month) bipolar and 60 (approximately 1 subject/month) healthy adolescents. Because within-subject contrasts minimize subject variance, we expected to observe greater intra-subject differences than those calculated for contrasts between groups. Based on our prior studies [1,2] we estimated that approximately 10% of patients would discontinue study participation prior to completing study procedures [3-5]. For the power calculations described, we considered large effect sizes of d > 0.8 to be clinically relevant. Treatment remission wat to be defined as having an endpoint YMRS score < 12 and a CDRS-R score < 28 and a CGI-S < 3. Although this definition of remission is based on those used in previous studies [6,7], since it is categorical, we also examined symptom rating changes as continuous measures for analyses.

**Treatment Procedures**

Following a clinical evaluation and MRI scanning, patients were randomized, by an investigational pharmacist, to double-blind treatment with quetiapine or lithium, and evaluated weekly for 6 weeks. The randomization schedule was stratified by presence vs. absence of ADHD, presence vs. absence of psychosis, and mood state (mixed vs. manic episode).

A total of 109 youth with bipolar I disorder were included. Of the 109 patients recruited, data from 9 were excluded at baseline due to failure to finish 3D T1 scan or excessive head motion during their 3D T1 scans. 12 patients in the lithium treatment group and 5 patients in the quetiapine treatment group were lost to follow-up due to one or more reasons including the following: a) moving out of town, b) lack of efficacy, c) withdrawal of consent, d) medication noncompliance, and 5 patients in the lithium treatment group and 4 patients in the quetiapine treatment group discontinued their drug intervention due to the adverse effects. Thus, analyses included 100 patients with bipolar disorder at baseline (55 patients that received quetiapine, and 45 patients who received lithium carbonate), 94 (53 patients receiving quetiapine and 41 patients receiving lithium carbonate) at week 1, and 74 (46 patients receiving quetiapine and 28 patients receiving lithium carbonate) at week 6 (see Table S1). Samples of healthy comparison youth included 63 at baseline, 59 at week 1 and 57 at week 6.

Quetiapine was initiated at 100 mg qhs and lithium carbonate was initiated at 30 mg/kg (maximum starting dose of 600 mg twice daily). Patients were also given placebo capsules for the medication to which they were not assigned. Quetiapine was titrated to a target dose of 400-600 mg/day based on tolerability and response. Lithium was titrated to a serum level of 1.0-1.2 mEq/L. Treatment was administered in a double-dummy, double-blind manner, with an unblinded study psychiatrist monitoring trough lithium levels and making dose adjustments independent from treating psychiatrist and clinical raters. Blinded clinical tolerability dose adjustment recommendations took precedent over un-blinded double dummy dose adjustment recommendations. There were no significant changes of the treatment methods or outcomes after trial commencement.

**Explanation of topological measurements used in current study**

A graph was formed by the basic elements ‘nodes’ and the connections ‘edges’ between them. Nodes connected by edges were called neighbors. For each node, we have three kinds of measurements describing the topological centralities including degree, betweenness and efficiency in current study. The most important network complexity measure is node degree since many other graph theory measures are related to it. The degree of a node equals to the number of edges connected to it. It reflects its information communication ability in the network. Betweenness centrality is defined as the fraction of all shortest paths in the network that pass through a given node, reflecting the nodes’ effects on information flow between other nodes. The efficiency of a node defined as the inverse of the shortest path length between one node and other nodes measuring how efficient information is transferred between this node and the others. The smaller the distance between nodes, the faster the information transfers. The average efficiency of all nodes in a graph is called global efficiency (E_g_). Similarly, the average of shortest path length of all nodes in a graph is called shortest path length of a graph (L_p_). Although both shortest path length and global efficiency are describing information transfer speed, global efficiency may be especially meaningful when considering the disconnected networks which have nodes don’t have any connection to other nodes in the graph, as paths between disconnected nodes are defined to have infinite path length and this situation can be easily characterized by zero efficiency [8]. Unlike global efficiency, the local efficiency of the network (E_loc_) measures how efficient communication is among the first neighbors of a given node when it is removed, reflecting the fault tolerant of the system [9]. Generally, small-world networks have high E_g_ and E_loc_ at the same time. Clustering coefficient (C_p_) describes the ability for functional segregation and efficiency of local information transfer. The clustering coefficient C_p_ at node p is a fraction of the number of existing connections between the neighbors of the node divided by the number of all possible connections of the graph. By definition, the E_loc_ plays a role similar to the C_p_. A network is a small-world network if it has a similar path length but greater clustering of nodes than an equivalent random graph which has the same number of nodes and edges. When we want to quantify the small-worldness of a network, we need to introduce normalized C_p_ (γ) and normalized L_p_ (λ). Normalized C_p_ is defined by the ratio between the clustering coefficients of actual and random graph, and normalized L_p_ is defined by the ratio between the shortest path length of actual and random graphs. Both normalized C_p_ and normalized L_p_ are have the same meaning as C_p_ and L_p_. The small-worldness (σ) metric is defined by the ratio between γ and λ. If σ > 1, this network can be viewed as a small-world network [10].

**Support vector machine model**

For responder and non-responder comparisons, we measured the performance of a support vector machine (SVM) [11] trained to classify responders vs. non-responders using the GM morphological matrices (90 × 90 network matrix). The SVM is a widely used machine learning model. We used the implementation from the Scikit-Learn library [12] that is based on LIBSVM [13]. It works as follows: first, the model maps the input data from the training set to the feature space using a set of mathematical functions known as kernels. Here, a linear kernel was preferred to a nonlinear kernel to minimize the risk of overfitting. In this feature space, the model learns the optimum separation surface that maximizes the margin between different classes. In our case the linear SVM has one hyperparameter (the soft margin parameter C), which affects the model’s training by controlling the trade-off between reducing training errors and increasing the separation margin. Once the separation surface is determined, it can be used to predict the class of new unseen observations.

To obtain a reliable estimate of the performance of the models, we used a 10-fold stratified cross-validation scheme. In this scheme the participants were divided into 10 non-overlapping partitions, each with the same proportion of patients and healthy controls. In each one of the ten iterations of the cross-validation, nine partitions were used as the training set to train the SVM model, and then the trained model was used to obtain predictions in the remaining partition. These predictions were used to calculate the performance metrics (balanced accuracy, specificity, and sensitivity), and since the test set was not part of the training process, the resulting values were unbiased. The reported performance in each case is the mean value across the cross-validation iterations. Finally, the statistical significance was estimated using the permutation method (1,000 permutations).

In each iteration of the cross-validation, we also performed a nested cross-validation inside the training set (i.e., 10-fold stratified nested cross-validation) to select the optimum C value for the SVM. This parameter was optimized by performing a grid search in the following range of values: C = 10^-3^, 10^-2^, 10^-1^, 1, 10, 10^2^, 10^3^, 10^4^. After selecting the best C value based on the balanced accuracy, an SVM was trained using the whole training set and used to assess performance on the test set. Note that the test set was not used during this hyperparameter search, to avoid biased results.

**References:**

1 DelBello MP, Goldman R, Phillips D, Deng L, Cucchiaro J, Loebel A. Efficacy and Safety of Lurasidone in Children and Adolescents With Bipolar I Depression: A Double-Blind, Placebo-Controlled Study. J Am Acad Child Adolesc Psychiatry. 2017;56(12):1015-25.

2 Findling RL, McNamara NK, Pavuluri M, Frazier JA, Rynn M, Scheffer R, et al. Lithium for the Maintenance Treatment of Bipolar I Disorder: A Double-Blind, Placebo-Controlled Discontinuation Study. J Am Acad Child Adolesc Psychiatry. 2019;58(2):287-96.e4.

3 Zhang W, Xiao Y, Sun H, Patino LR, Tallman MJ, Weber WA, et al. Discrete patterns of cortical thickness in youth with bipolar disorder differentially predict treatment response to quetiapine but not lithium. Neuropsychopharmacology. 2018;43(11):2256-63.

4 Nery FG, Norris M, Eliassen JC, Weber WA, Blom TJ, Welge JA, et al. White matter volumes in youth offspring of bipolar parents. J Affect Disord. 2017;209:246-53.

5 Welge JA, Saliba LJ, Strawn JR, Eliassen JC, Patino LR, Adler CM, et al. Neurofunctional Differences Among Youth With and at Varying Risk for Developing Mania. J Am Acad Child Adolesc Psychiatry. 2016;55(11):980-89.

6 Patel NC, Patrick DM, Youngstrom EA, Strakowski SM, Delbello MP. Response and remission in adolescent mania: signal detection analyses of the young mania rating scale. J Am Acad Child Adolesc Psychiatry. 2007;46(5):628-35.

7 Ketter TA, Jones M, Paulsson B. Rates of remission/euthymia with quetiapine monotherapy compared with placebo in patients with acute mania. J Affect Disord. 2007;100 Suppl 1:S45-53.

8 Rubinov M, Sporns O. Complex network measures of brain connectivity: uses and interpretations. Neuroimage. 2010;52(3):1059-69.

9 Latora V, Marchiori M. Efficient behavior of small-world networks. Phys Rev Lett. 2001;87(19):198701.

10 Humphries MD, Gurney K. Network 'small-world-ness': a quantitative method for determining canonical network equivalence. PLoS One. 2008;3(4):e0002051.

11 Cortes C, Vapnik V. Support Vector Network. Machine learning. 1995;20(3):273-97.

12 Pedregosa F, Varoquaux G, Gramfort A, Michel V, Thirion B, Grisel O, et al. Scikit-learn: Machine Learning in Python. Journal of Machine Learning Research. 2012;12(10):2825-30.

13 Chang CC, Lin CJ. LIBSVM: A library for support vector machines. Acm Transactions on Intelligent Systems & Technology. 2011;2(3):1-27.

| *Subgroup* | **Lithium** | **Quetiapin**e |  |
| --- | --- | --- | --- |
| *Subgroup sample size* | 45 | 55 | *Lithium vs Quetiapine* |
| YMRS (*baseline*) | 28.02$\pm$5.58 | 27.69$\pm$5.37 | *p =0.76* ^a^ |
| YMRS (*end point*) | 12.40$\pm$7.42 | 9.24$\pm$6.27 | *p =0.07* ^a^ |
| CDRS-R*(baseline)* | 38.62$\pm$7.97 | 37.33$\pm$9.01 | *p=0.45* ^a^ |
| CDRS-R(*end point*) | 27.15$\pm$6.70 | 25.85$\pm$7.22 | *p=0.46* ^a^ |
| Dosage (mg) mean(SD) | 1304.44$\pm$275.48 | 496.36$\pm$83.81 |  |
| *Longitudinal changes in combined bipolar groups* | |  |  |
| YMRS (*baseline*) 27.84$\pm$6.90 vs YMRS (*endpoint*) 9.84$\pm$6. 40 | | | *p* < 0.001 ^a^ |
| CDRS-R *(baseline)* 37.91$\pm$8.54 vs CDRS-R (*endpoint*) 26.36$\pm$7.00 | | | *p* < 0.001 ^a^ |

**Table S1.** Clinical characteristics of youth with Bipolar Disorder.

Data are presented as the range of the mean $\pm$SD. YMRS Young Mania Rating Scale, CDRS-R = Children’s Depression Rating Scale-Revised, SD = standard deviation.

^a^ The *p* value was calculated by Welch two sample t test.

**Table S2.** Intraclass correlation coefficients among healthy individuals for all topological measurements used in current study.

| Measurements | Regions | ICC value | ICC lbound | ICC ubound |
| --- | --- | --- | --- | --- |
| ***Global*** |  | 0.60 | 0.45 | 0.72 |
| Global Efficiency |  | 0.61 | 0.47 | 0.73 |
| Local Efficiency |  | 0.56 | 0.41 | 0.69 |
| Clustering Coefficients |  | 0.57 | 0.42 | 0.70 |
| γ |  | 0.25 | 0.09 | 0.43 |
| λ |  | 0.58 | 0.43 | 0.71 |
| Shortest Path Length |  | 0.57 | 0.42 | 0.70 |
| σ |  | 0.49 | 0.33 | 0.64 |
| ***Regional*** |  | 0.42 | 0.25 | 0.58 |
| Betweenness | Precentral gyrus L | 0.40 | 0.23 | 0.56 |
| Betweenness | Precentral gyrus R | 0.26 | 0.09 | 0.44 |
| Betweenness | Superior frontal gyrus, dorsolateral, L | 0.30 | 0.14 | 0.48 |
| Betweenness | Superior frontal gyrus, dorsolateral, R | 0.54 | 0.39 | 0.68 |
| Betweenness | Superior frontal gyrus, orbital part, L | 0.39 | 0.22 | 0.55 |
| Betweenness | Superior frontal gyrus, orbital part, R | 0.45 | 0.28 | 0.60 |
| Betweenness | Middle frontal gyrus, L | 0.42 | 0.26 | 0.58 |
| Betweenness | Middle frontal gyrus, R | 0.41 | 0.24 | 0.57 |
| Betweenness | Middle frontal gyrus, orbital part, L | 0.44 | 0.27 | 0.60 |
| Betweenness | Middle frontal gyrus, orbital part, R | 0.51 | 0.35 | 0.65 |
| Betweenness | Inferior frontal gyrus, opercular part, L | 0.59 | 0.44 | 0.72 |
| Betweenness | Inferior frontal gyrus, opercular part, R | 0.46 | 0.29 | 0.61 |
| Betweenness | Inferior frontal gyrus, triangular part, L | 0.38 | 0.22 | 0.55 |
| Betweenness | Inferior frontal gyrus, triangular part, R | 0.31 | 0.14 | 0.48 |
| Betweenness | Inferior frontal gyrus, orbital part, L | 0.51 | 0.36 | 0.66 |
| Betweenness | Inferior frontal gyrus, orbital part, R | 0.64 | 0.51 | 0.76 |
| Betweenness | Rolandic operculum, L | 0.49 | 0.33 | 0.64 |
| Betweenness | Rolandic operculum, R | 0.44 | 0.27 | 0.59 |
| Betweenness | Supplementary motor area, L | 0.38 | 0.21 | 0.54 |
| Betweenness | Supplementary motor area, R | 0.21 | 0.05 | 0.39 |
| Betweenness | Olfactory cortex, L | 0.45 | 0.29 | 0.61 |
| Betweenness | Olfactory cortex, R | 0.63 | 0.50 | 0.75 |
| Betweenness | Superior frontal gyrus, medial, L | 0.24 | 0.08 | 0.42 |
| Betweenness | Superior frontal gyrus, medial, R | 0.38 | 0.21 | 0.55 |
| Betweenness | Superior frontal gyrus, medial orbital, L | 0.34 | 0.18 | 0.51 |
| Betweenness | Superior frontal gyrus, medial orbital, R | 0.31 | 0.14 | 0.49 |
| Betweenness | Gyrus rectus, L | 0.58 | 0.44 | 0.71 |
| Betweenness | Gyrus rectus, R | 0.19 | 0.03 | 0.37 |
| Betweenness | Insula, L | 0.22 | 0.05 | 0.39 |
| Betweenness | Insula, R | 0.40 | 0.23 | 0.56 |
| Betweenness | Anterior cingulate and paracingulate gyri, L | 0.35 | 0.19 | 0.52 |
| Betweenness | Anterior cingulate and paracingulate gyri, R | 0.33 | 0.16 | 0.50 |
| Betweenness | Median cingulate and paracingulate gyri, L | 0.57 | 0.43 | 0.71 |
| Betweenness | Median cingulate and paracingulate gyri, R | NA | NA | NA |
| Betweenness | Posterior cingulate gyrus, L | 0.39 | 0.22 | 0.55 |
| Betweenness | Posterior cingulate gyrus, R | 0.71 | 0.59 | 0.80 |
| Betweenness | Hippocampus, L | 0.38 | 0.21 | 0.54 |
| Betweenness | Hippocampus, R | 0.30 | 0.13 | 0.47 |
| Betweenness | Parahippocampal gyrus, L | 0.27 | 0.10 | 0.44 |
| Betweenness | Parahippocampal gyrus, R | 0.17 | 0.01 | 0.35 |
| Betweenness | Amygdala, L | 0.36 | 0.20 | 0.53 |
| Betweenness | Amygdala, R | 0.45 | 0.29 | 0.61 |
| Betweenness | Calcarine fissure and surrounding cortex, L | 0.51 | 0.35 | 0.65 |
| Betweenness | Calcarine fissure and surrounding cortex, R | 0.49 | 0.33 | 0.64 |
| Betweenness | Cuneus, L | 0.29 | 0.12 | 0.47 |
| Betweenness | Cuneus, R | 0.37 | 0.20 | 0.53 |
| Betweenness | Lingual gyrus, L | 0.56 | 0.41 | 0.70 |
| Betweenness | Lingual gyrus, R | 0.62 | 0.47 | 0.74 |
| Betweenness | Superior occipital gyrus, L | 0.35 | 0.18 | 0.52 |
| Betweenness | Superior occipital gyrus, R | 0.48 | 0.32 | 0.63 |
| Betweenness | Middle occipital gyrus, L | 0.33 | 0.16 | 0.50 |
| Betweenness | Middle occipital gyrus, R | 0.28 | 0.11 | 0.45 |
| Betweenness | Inferior occipital gyrus, L | 0.62 | 0.47 | 0.74 |
| Betweenness | Inferior occipital gyrus, R | 0.42 | 0.25 | 0.58 |
| Betweenness | Fusiform gyrus, L | 0.20 | 0.04 | 0.38 |
| Betweenness | Fusiform gyrus, R | 0.26 | 0.10 | 0.44 |
| Betweenness | Postcentral gyrus, L | 0.42 | 0.25 | 0.58 |
| Betweenness | Postcentral gyrus, R | 0.09 | -0.06 | 0.27 |
| Betweenness | Superior parietal gyrus, L | 0.37 | 0.21 | 0.54 |
| Betweenness | Superior parietal gyrus, R | 0.36 | 0.20 | 0.53 |
| Betweenness | Inferior parietal, but supramarginal and angular gyri, L | 0.47 | 0.31 | 0.62 |
| Betweenness | Inferior parietal, but supramarginal and angular gyri, R | 0.50 | 0.34 | 0.65 |
| Betweenness | Supramarginal gyrus, L | 0.30 | 0.13 | 0.47 |
| Betweenness | Supramarginal gyrus, R | 0.42 | 0.25 | 0.58 |
| Betweenness | Angular gyrus, L | 0.64 | 0.50 | 0.75 |
| Betweenness | Angular gyrus, R | 0.46 | 0.29 | 0.61 |
| Betweenness | Precuneus, L | 0.24 | 0.08 | 0.42 |
| Betweenness | Precuneus, R | 0.48 | 0.32 | 0.63 |
| Betweenness | Paracentral lobule, L | 0.66 | 0.53 | 0.77 |
| Betweenness | Paracentral lobule, R | 0.57 | 0.42 | 0.70 |
| Betweenness | Caudate nucleus, L | 0.12 | -0.03 | 0.30 |
| Betweenness | Caudate nucleus, R | 0.33 | 0.16 | 0.50 |
| Betweenness | Lenticular nucleus putamen, L | 0.49 | 0.34 | 0.64 |
| Betweenness | Lenticular nucleus putamen, R | 0.17 | 0.01 | 0.35 |
| Betweenness | Lenticular nucleus, pallidum, L | 0.03 | -0.11 | 0.21 |
| Betweenness | Lenticular nucleus, pallidum, R | 0.60 | 0.46 | 0.73 |
| Betweenness | Thalamus, L | 0.13 | -0.03 | 0.31 |
| Betweenness | Thalamus, R | 0.23 | 0.06 | 0.41 |
| Betweenness | Heschl gyrus, L | 0.45 | 0.28 | 0.60 |
| Betweenness | Heschl gyrus, R | 0.67 | 0.54 | 0.78 |
| Betweenness | Superior temporal gyrus, L | 0.40 | 0.23 | 0.56 |
| Betweenness | Superior temporal gyrus, R | 0.43 | 0.27 | 0.59 |
| Betweenness | Temporal pole: superior temporal gyrus, L | 0.52 | 0.36 | 0.66 |
| Betweenness | Temporal pole: superior temporal gyrus, R | 0.53 | 0.37 | 0.67 |
| Betweenness | Middle temporal gyrus, L | 0.50 | 0.34 | 0.64 |
| Betweenness | Middle temporal gyrus, R | 0.15 | -0.01 | 0.33 |
| Betweenness | Temporal pole: middle temporal gyrus, L | 0.31 | 0.14 | 0.48 |
| Betweenness | Temporal pole: middle temporal gyrus, R | 0.28 | 0.12 | 0.46 |
| Betweenness | Inferior temporal gyrus, L | 0.79 | 0.70 | 0.86 |
| Betweenness | Inferior temporal gyrus, R | 0.71 | 0.59 | 0.80 |
| Degree | Precentral gyrus L | 0.71 | 0.59 | 0.81 |
| Degree | Precentral gyrus R | 0.72 | 0.61 | 0.82 |
| Degree | Superior frontal gyrus, dorsolateral, L | 0.64 | 0.51 | 0.76 |
| Degree | Superior frontal gyrus, dorsolateral, R | 0.57 | 0.43 | 0.71 |
| Degree | Superior frontal gyrus, orbital part, L | 0.72 | 0.60 | 0.81 |
| Degree | Superior frontal gyrus, orbital part, R | 0.68 | 0.56 | 0.79 |
| Degree | Middle frontal gyrus, L | 0.69 | 0.56 | 0.79 |
| Degree | Middle frontal gyrus, R | 0.67 | 0.54 | 0.78 |
| Degree | Middle frontal gyrus, orbital part, L | 0.80 | 0.71 | 0.87 |
| Degree | Middle frontal gyrus, orbital part, R | 0.72 | 0.60 | 0.81 |
| Degree | Inferior frontal gyrus, opercular part, L | 0.88 | 0.82 | 0.92 |
| Degree | Inferior frontal gyrus, opercular part, R | 0.69 | 0.57 | 0.80 |
| Degree | Inferior frontal gyrus, triangular part, L | 0.64 | 0.51 | 0.76 |
| Degree | Inferior frontal gyrus, triangular part, R | 0.60 | 0.46 | 0.73 |
| Degree | Inferior frontal gyrus, orbital part, L | 0.76 | 0.66 | 0.85 |
| Degree | Inferior frontal gyrus, orbital part, R | 0.80 | 0.71 | 0.87 |
| Degree | Rolandic operculum, L | 0.61 | 0.46 | 0.73 |
| Degree | Rolandic operculum, R | 0.73 | 0.62 | 0.82 |
| Degree | Supplementary motor area, L | 0.64 | 0.50 | 0.75 |
| Degree | Supplementary motor area, R | 0.45 | 0.29 | 0.61 |
| Degree | Olfactory cortex, L | 0.71 | 0.59 | 0.81 |
| Degree | Olfactory cortex, R | 0.81 | 0.72 | 0.88 |
| Degree | Superior frontal gyrus, medial, L | 0.58 | 0.44 | 0.71 |
| Degree | Superior frontal gyrus, medial, R | 0.73 | 0.61 | 0.82 |
| Degree | Superior frontal gyrus, medial orbital, L | 0.59 | 0.45 | 0.72 |
| Degree | Superior frontal gyrus, medial orbital, R | 0.51 | 0.35 | 0.65 |
| Degree | Gyrus rectus, L | 0.84 | 0.76 | 0.90 |
| Degree | Gyrus rectus, R | 0.71 | 0.59 | 0.80 |
| Degree | Insula, L | 0.82 | 0.74 | 0.88 |
| Degree | Insula, R | 0.78 | 0.69 | 0.86 |
| Degree | Anterior cingulate and paracingulate gyri, L | 0.66 | 0.53 | 0.77 |
| Degree | Anterior cingulate and paracingulate gyri, R | 0.79 | 0.69 | 0.86 |
| Degree | Median cingulate and paracingulate gyri, L | 0.72 | 0.60 | 0.81 |
| Degree | Median cingulate and paracingulate gyri, R | 0.55 | 0.40 | 0.69 |
| Degree | Posterior cingulate gyrus, L | 0.64 | 0.50 | 0.75 |
| Degree | Posterior cingulate gyrus, R | 0.55 | 0.40 | 0.69 |
| Degree | Hippocampus, L | 0.67 | 0.54 | 0.78 |
| Degree | Hippocampus, R | 0.55 | 0.40 | 0.69 |
| Degree | Parahippocampal gyrus, L | 0.73 | 0.62 | 0.82 |
| Degree | Parahippocampal gyrus, R | 0.42 | 0.26 | 0.58 |
| Degree | Amygdala, L | 0.72 | 0.61 | 0.82 |
| Degree | Amygdala, R | 0.72 | 0.60 | 0.81 |
| Degree | Calcarine fissure and surrounding cortex, L | 0.89 | 0.84 | 0.93 |
| Degree | Calcarine fissure and surrounding cortex, R | 0.85 | 0.77 | 0.90 |
| Degree | Cuneus, L | 0.75 | 0.65 | 0.84 |
| Degree | Cuneus, R | 0.72 | 0.61 | 0.82 |
| Degree | Lingual gyrus, L | 0.86 | 0.80 | 0.91 |
| Degree | Lingual gyrus, R | 0.76 | 0.65 | 0.84 |
| Degree | Superior occipital gyrus, L | 0.69 | 0.57 | 0.80 |
| Degree | Superior occipital gyrus, R | 0.77 | 0.67 | 0.85 |
| Degree | Middle occipital gyrus, L | 0.61 | 0.47 | 0.73 |
| Degree | Middle occipital gyrus, R | 0.59 | 0.45 | 0.72 |
| Degree | Inferior occipital gyrus, L | 0.79 | 0.69 | 0.86 |
| Degree | Inferior occipital gyrus, R | 0.67 | 0.54 | 0.78 |
| Degree | Fusiform gyrus, L | 0.58 | 0.43 | 0.71 |
| Degree | Fusiform gyrus, R | 0.71 | 0.60 | 0.81 |
| Degree | Postcentral gyrus, L | 0.78 | 0.68 | 0.85 |
| Degree | Postcentral gyrus, R | 0.73 | 0.62 | 0.82 |
| Degree | Superior parietal gyrus, L | 0.84 | 0.77 | 0.90 |
| Degree | Superior parietal gyrus, R | 0.65 | 0.51 | 0.76 |
| Degree | Inferior parietal, but supramarginal and angular gyri, L | 0.72 | 0.60 | 0.81 |
| Degree | Inferior parietal, but supramarginal and angular gyri, R | 0.79 | 0.70 | 0.87 |
| Degree | Supramarginal gyrus, L | 0.69 | 0.57 | 0.79 |
| Degree | Supramarginal gyrus, R | 0.70 | 0.58 | 0.80 |
| Degree | Angular gyrus, L | 0.81 | 0.73 | 0.88 |
| Degree | Angular gyrus, R | 0.79 | 0.70 | 0.86 |
| Degree | Precuneus, L | 0.75 | 0.64 | 0.83 |
| Degree | Precuneus, R | 0.68 | 0.55 | 0.78 |
| Degree | Paracentral lobule, L | 0.81 | 0.72 | 0.88 |
| Degree | Paracentral lobule, R | 0.88 | 0.82 | 0.93 |
| Degree | Caudate nucleus, L | 0.52 | 0.36 | 0.66 |
| Degree | Caudate nucleus, R | 0.54 | 0.39 | 0.68 |
| Degree | Lenticular nucleus putamen, L | 0.80 | 0.71 | 0.87 |
| Degree | Lenticular nucleus putamen, R | 0.51 | 0.35 | 0.65 |
| Degree | Lenticular nucleus, pallidum, L | 0.46 | 0.29 | 0.61 |
| Degree | Lenticular nucleus, pallidum, R | 0.59 | 0.44 | 0.72 |
| Degree | Thalamus, L | 0.60 | 0.46 | 0.73 |
| Degree | Thalamus, R | 0.64 | 0.50 | 0.75 |
| Degree | Heschl gyrus, L | 0.79 | 0.70 | 0.87 |
| Degree | Heschl gyrus, R | 0.75 | 0.65 | 0.84 |
| Degree | Superior temporal gyrus, L | 0.61 | 0.47 | 0.74 |
| Degree | Superior temporal gyrus, R | 0.46 | 0.30 | 0.61 |
| Degree | Temporal pole: superior temporal gyrus, L | 0.70 | 0.58 | 0.80 |
| Degree | Temporal pole: superior temporal gyrus, R | 0.77 | 0.66 | 0.85 |
| Degree | Middle temporal gyrus, L | 0.53 | 0.38 | 0.67 |
| Degree | Middle temporal gyrus, R | 0.46 | 0.29 | 0.61 |
| Degree | Temporal pole: middle temporal gyrus, L | 0.61 | 0.47 | 0.73 |
| Degree | Temporal pole: middle temporal gyrus, R | 0.49 | 0.33 | 0.64 |
| Degree | Inferior temporal gyrus, L | 0.77 | 0.67 | 0.85 |
| Degree | Inferior temporal gyrus, R | 0.72 | 0.60 | 0.81 |
| Node Efficiency | Precentral gyrus L | 0.69 | 0.56 | 0.79 |
| Node Efficiency | Precentral gyrus R | 0.72 | 0.60 | 0.81 |
| Node Efficiency | Superior frontal gyrus, dorsolateral, L | 0.62 | 0.48 | 0.74 |
| Node Efficiency | Superior frontal gyrus, dorsolateral, R | 0.60 | 0.45 | 0.72 |
| Node Efficiency | Superior frontal gyrus, orbital part, L | 0.74 | 0.63 | 0.83 |
| Node Efficiency | Superior frontal gyrus, orbital part, R | 0.68 | 0.55 | 0.78 |
| Node Efficiency | Middle frontal gyrus, L | 0.71 | 0.60 | 0.81 |
| Node Efficiency | Middle frontal gyrus, R | 0.69 | 0.56 | 0.79 |
| Node Efficiency | Middle frontal gyrus, orbital part, L | 0.81 | 0.72 | 0.88 |
| Node Efficiency | Middle frontal gyrus, orbital part, R | 0.74 | 0.63 | 0.83 |
| Node Efficiency | Inferior frontal gyrus, opercular part, L | 0.88 | 0.82 | 0.92 |
| Node Efficiency | Inferior frontal gyrus, opercular part, R | 0.59 | 0.44 | 0.72 |
| Node Efficiency | Inferior frontal gyrus, triangular part, L | 0.65 | 0.51 | 0.76 |
| Node Efficiency | Inferior frontal gyrus, triangular part, R | 0.62 | 0.47 | 0.74 |
| Node Efficiency | Inferior frontal gyrus, orbital part, L | 0.73 | 0.62 | 0.82 |
| Node Efficiency | Inferior frontal gyrus, orbital part, R | 0.76 | 0.66 | 0.84 |
| Node Efficiency | Rolandic operculum, L | 0.62 | 0.48 | 0.74 |
| Node Efficiency | Rolandic operculum, R | 0.77 | 0.67 | 0.85 |
| Node Efficiency | Supplementary motor area, L | 0.60 | 0.46 | 0.73 |
| Node Efficiency | Supplementary motor area, R | 0.51 | 0.36 | 0.66 |
| Node Efficiency | Olfactory cortex, L | 0.75 | 0.64 | 0.84 |
| Node Efficiency | Olfactory cortex, R | 0.83 | 0.76 | 0.89 |
| Node Efficiency | Superior frontal gyrus, medial, L | 0.54 | 0.38 | 0.68 |
| Node Efficiency | Superior frontal gyrus, medial, R | 0.75 | 0.64 | 0.83 |
| Node Efficiency | Superior frontal gyrus, medial orbital, L | 0.53 | 0.38 | 0.67 |
| Node Efficiency | Superior frontal gyrus, medial orbital, R | 0.46 | 0.29 | 0.61 |
| Node Efficiency | Gyrus rectus, L | 0.82 | 0.73 | 0.88 |
| Node Efficiency | Gyrus rectus, R | 0.70 | 0.58 | 0.80 |
| Node Efficiency | Insula, L | 0.81 | 0.72 | 0.88 |
| Node Efficiency | Insula, R | 0.78 | 0.68 | 0.86 |
| Node Efficiency | Anterior cingulate and paracingulate gyri, L | 0.68 | 0.55 | 0.79 |
| Node Efficiency | Anterior cingulate and paracingulate gyri, R | 0.78 | 0.69 | 0.86 |
| Node Efficiency | Median cingulate and paracingulate gyri, L | 0.64 | 0.50 | 0.76 |
| Node Efficiency | Median cingulate and paracingulate gyri, R | 0.67 | 0.54 | 0.78 |
| Node Efficiency | Posterior cingulate gyrus, L | 0.62 | 0.48 | 0.74 |
| Node Efficiency | Posterior cingulate gyrus, R | 0.48 | 0.32 | 0.63 |
| Node Efficiency | Hippocampus, L | 0.64 | 0.51 | 0.76 |
| Node Efficiency | Hippocampus, R | 0.49 | 0.33 | 0.64 |
| Node Efficiency | Parahippocampal gyrus, L | 0.75 | 0.65 | 0.84 |
| Node Efficiency | Parahippocampal gyrus, R | 0.42 | 0.26 | 0.58 |
| Node Efficiency | Amygdala, L | 0.75 | 0.64 | 0.83 |
| Node Efficiency | Amygdala, R | 0.73 | 0.61 | 0.82 |
| Node Efficiency | Calcarine fissure and surrounding cortex, L | 0.89 | 0.83 | 0.93 |
| Node Efficiency | Calcarine fissure and surrounding cortex, R | 0.86 | 0.80 | 0.91 |
| Node Efficiency | Cuneus, L | 0.74 | 0.63 | 0.83 |
| Node Efficiency | Cuneus, R | 0.72 | 0.60 | 0.81 |
| Node Efficiency | Lingual gyrus, L | 0.86 | 0.79 | 0.91 |
| Node Efficiency | Lingual gyrus, R | 0.77 | 0.67 | 0.85 |
| Node Efficiency | Superior occipital gyrus, L | 0.67 | 0.54 | 0.77 |
| Node Efficiency | Superior occipital gyrus, R | 0.79 | 0.70 | 0.87 |
| Node Efficiency | Middle occipital gyrus, L | 0.60 | 0.46 | 0.73 |
| Node Efficiency | Middle occipital gyrus, R | 0.62 | 0.48 | 0.74 |
| Node Efficiency | Inferior occipital gyrus, L | 0.77 | 0.66 | 0.85 |
| Node Efficiency | Inferior occipital gyrus, R | 0.70 | 0.57 | 0.80 |
| Node Efficiency | Fusiform gyrus, L | 0.61 | 0.46 | 0.73 |
| Node Efficiency | Fusiform gyrus, R | 0.61 | 0.47 | 0.73 |
| Node Efficiency | Postcentral gyrus, L | 0.80 | 0.71 | 0.87 |
| Node Efficiency | Postcentral gyrus, R | 0.62 | 0.48 | 0.74 |
| Node Efficiency | Superior parietal gyrus, L | 0.86 | 0.80 | 0.91 |
| Node Efficiency | Superior parietal gyrus, R | 0.68 | 0.55 | 0.79 |
| Node Efficiency | Inferior parietal, but supramarginal and angular gyri, L | 0.72 | 0.61 | 0.82 |
| Node Efficiency | Inferior parietal, but supramarginal and angular gyri, R | 0.81 | 0.72 | 0.88 |
| Node Efficiency | Supramarginal gyrus, L | 0.71 | 0.59 | 0.81 |
| Node Efficiency | Supramarginal gyrus, R | 0.75 | 0.64 | 0.83 |
| Node Efficiency | Angular gyrus, L | 0.83 | 0.74 | 0.89 |
| Node Efficiency | Angular gyrus, R | 0.86 | 0.79 | 0.91 |
| Node Efficiency | Precuneus, L | 0.69 | 0.57 | 0.80 |
| Node Efficiency | Precuneus, R | 0.70 | 0.57 | 0.80 |
| Node Efficiency | Paracentral lobule, L | 0.83 | 0.75 | 0.89 |
| Node Efficiency | Paracentral lobule, R | 0.90 | 0.85 | 0.94 |
| Node Efficiency | Caudate nucleus, L | 0.51 | 0.35 | 0.65 |
| Node Efficiency | Caudate nucleus, R | 0.56 | 0.41 | 0.70 |
| Node Efficiency | Lenticular nucleus putamen, L | 0.83 | 0.75 | 0.89 |
| Node Efficiency | Lenticular nucleus putamen, R | 0.67 | 0.54 | 0.78 |
| Node Efficiency | Lenticular nucleus, pallidum, L | 0.22 | 0.05 | 0.40 |
| Node Efficiency | Lenticular nucleus, pallidum, R | 0.54 | 0.38 | 0.68 |
| Node Efficiency | Thalamus, L | 0.60 | 0.45 | 0.72 |
| Node Efficiency | Thalamus, R | 0.55 | 0.40 | 0.69 |
| Node Efficiency | Heschl gyrus, L | 0.81 | 0.72 | 0.87 |
| Node Efficiency | Heschl gyrus, R | 0.76 | 0.65 | 0.84 |
| Node Efficiency | Superior temporal gyrus, L | 0.58 | 0.44 | 0.71 |
| Node Efficiency | Superior temporal gyrus, R | 0.49 | 0.33 | 0.64 |
| Node Efficiency | Temporal pole: superior temporal gyrus, L | 0.68 | 0.56 | 0.79 |
| Node Efficiency | Temporal pole: superior temporal gyrus, R | 0.76 | 0.65 | 0.84 |
| Node Efficiency | Middle temporal gyrus, L | 0.41 | 0.25 | 0.57 |
| Node Efficiency | Middle temporal gyrus, R | 0.46 | 0.30 | 0.62 |
| Node Efficiency | Temporal pole: middle temporal gyrus, L | 0.58 | 0.44 | 0.71 |
| Node Efficiency | Temporal pole: middle temporal gyrus, R | 0.48 | 0.32 | 0.63 |
| Node Efficiency | Inferior temporal gyrus, L | 0.60 | 0.45 | 0.72 |
| Node Efficiency | Inferior temporal gyrus, R | 0.61 | 0.47 | 0.73 |

Abbreviation: L, left; R, right; lbound, lower bound of 95% confidence interval; ubound, upper bound of 95% confidence interval.

Table S3. Top 10 most relevant brain regions contributing to the SVM classification

| Lithium -treated group | Quetiapine- treated group | All Bipolar |
| --- | --- | --- |
| Superior frontal gyrus, medial L | Inferior frontal gyrus, orbital part L | Inferior frontal gyrus, orbital part L |
| Insula L | Middle temporal gyrus L | Paracentral lobule L |
| Heschl gyrus L | Inferior parietal gyrus R | Superior frontal gyrus, orbital part R |
| Angular gyrus R | Insula L | Middle temporal gyrus L |
| Cuneus R | Paracentral lobule R | Inferior frontal gyrus,opercular part R |
| Inferior temporal gyrus L | Calcarine R | Superior occipital gyrus R |
| Middle frontal gyrus, orbital part R | Inferior temporal gyrus L | Inferior parietal gyrus R |
| Precentral gyrus L | Superior occipital gyrus R | Insula L |
| Gyrus rectus L | Putamen R | Putamen R |
| Superior frontal gyrus, orbital part R | Olfactory cortex L | Inferior temporal gyrus L |

All the brain regions are from AAL (automated anatomical labeling).

Abbreviation: R: right, L: left. SVM: support vector machine.





Figure S1: distribution of YMRS at baseline (A), YMRS changes from baseline to week 1 (B) and YMRS changes from baseline to week 6 (C). YMRS, Young Mania Rating Scores; Wk1, week 1; Wk6, Week 6.
