# Supplementary material for: Changes in the brain structural connectome after a prospective randomized clinical trial of lithium and quetiapine treatment in youth with bipolar disorder
Source: Neuropsychopharmacology. 2021 Mar 22;46(7):1315–23. doi: 10.1038/s41386-021-00989-5 (PMC8134458; doi:10.1038/s41386-021-00989-5)
Supplement: Supplementary file 2 — CONSORT 2010 Flow Diagram [file 41386_2021_989_MOESM2_ESM.doc]

**
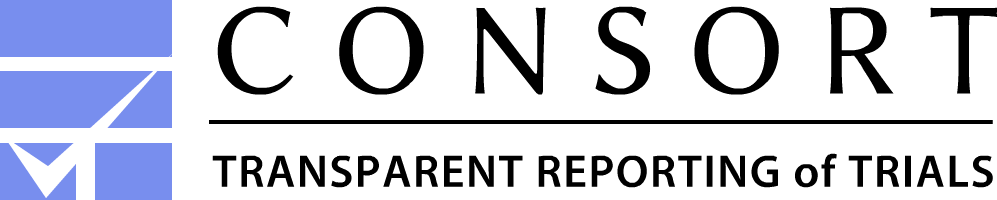
**

**CONSORT 2010 Flow Diagram**

**Allocation**

**Analysis**

**Follow-Up**

**Enrollment**

Assessed for eligibility (n=109)

Excluded (n=9)

♦  Not meeting inclusion criteria (n=9)

♦  Declined to participate (n=0)

♦  Other reasons (n=0)

Analysed (n=45 for baseline and n=28 for longitudinal analysis)
♦ Excluded from analysis (give reasons) (n=0)

Lost to follow-up (a. moving out of town b. lack of efficacy c. withdrawal of consent d. medication noncompliance) (n=12)

Discontinued intervention (adverse events) (n=5)

Allocated to intervention: treated with lithium (n=45)

♦ Received allocated intervention (n=45)

♦ Did not receive allocated intervention (give reasons) (n=0 )

Lost to follow-up (a. moving out of town b. lack of efficacy c. withdrawal of consent d. medication noncompliance) (n=5)

Discontinued intervention (adverse events) (n=4)

Allocated to intervention: treated with quetiapine (n=55)

♦ Received allocated intervention (n= 55)

♦ Did not receive allocated intervention (give reasons) (n=0)

Analysed (n=55 for baseline and n=46 for longitudinal analysis)
♦ Excluded from analysis (give reasons) (n=0)

Randomized (n=100)
